# Severe Acute Respiratory Syndrome Coronavirus 2 Induced Focal Segmental Glomerulosclerosis

**DOI:** 10.7759/cureus.10898

**Published:** 2020-10-11

**Authors:** Is-haq O Malik, Nida Ladiwala, Siddharth Chinta, Muhammad Khan, Komal Patel

**Affiliations:** 1 Internal Medicine, BronxCare Health System, Bronx, USA

**Keywords:** sars-cov-2, covid-19, collapsing fsgs, fsgs, acute kidney injury, proteinuria, esrd, apol1 gene, severe acute respiratory syndrome coronavirus 2, focal segmental glomerulosclerosis

## Abstract

Focal segmental glomerulosclerosis (FSGS) is a common cause of nephrotic syndrome affecting adults and children. Collapsing focal segmental glomerulosclerosis (FSGS), one of five histologic variants of FSGS is described as segmental or global collapse and sclerosis of the glomerular tufts and has been frequently associated with human immunodeficiency virus-associated nephropathy (HIVAN). Its association with other viral and non-viral causes, medications and other disease states has since been established. Due to its resistance to therapy, rapid progression to end-stage renal disease (ESRD) and overall poorer prognosis, identification with electron microscopy examination of the kidney biopsy sample is required during evaluation.

## Introduction

The novel severe acute respiratory syndrome coronavirus 2 (SARS-CoV-2) has spread globally, with approximately 11 million affected patients and upwards of 500,000 deaths at the time of writing. The disease spread through respiratory droplets and via contact causing pneumonia with multi-organ disease. Though the pathophysiology of coronavirus disease 2019 (COVID-19) causing multi-organ failure has not been fully understood, the interaction between angiotensin-converting enzyme 2 (ACE-2) and SARS family of viruses has been identified as a factor facilitating the infectivity of the virus. Given the abundance of ACE-2 receptors in lungs, pneumonia, and respiratory failure are predominant presentations in symptomatic patients. Initial reports showed acute kidney injury in up to 9% of patients with COVID-19 with acute tubular injury as the underlying pathophysiological mechanism [1]. However, subsequent studies noted up to 15% prevalence of kidney injury, with hematuria and proteinuria observed at 26% and 44% respectively [2-4]. This paved the way to pursue a kidney biopsy to understand and categorize the mechanism of injury. Presence of chronic kidney disease at baseline has shown to increase mortality risk in patients with COVID-19. We, therefore, present a patient with COVID-19 who developed acute renal failure caused by biopsy-proven collapsing focal segmental glomerulosclerosis (FSGS).

## Case presentation

**Figure 1:**
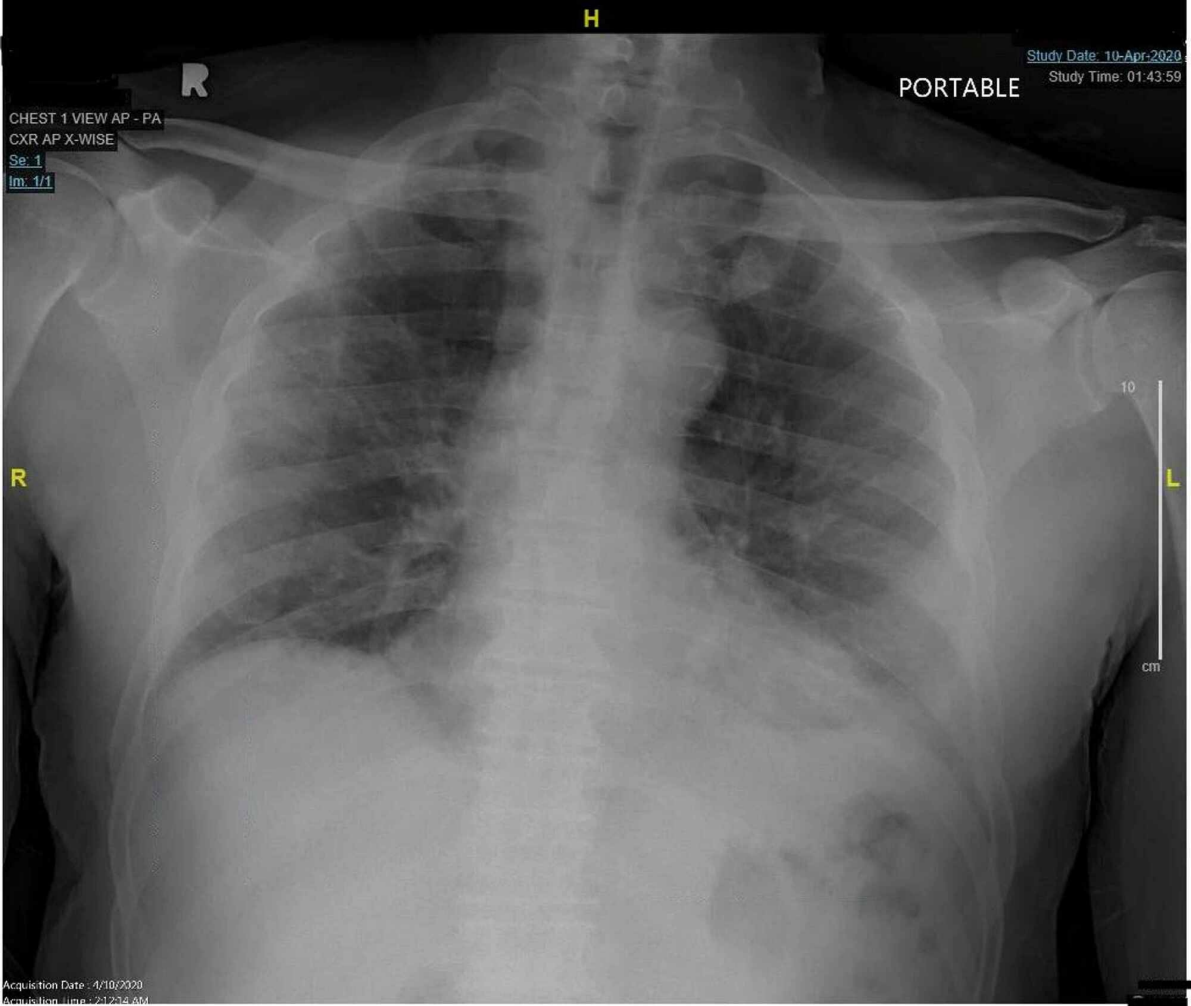
Chest x-ray on admission

We present a 57-year-old African-American male patient who was initially admitted in April with dizziness, diarrhea and hypoxia. The patient had additional symptoms of lymphopenia, elevated inflammatory markers (Table 1) and bilateral patchy infiltrates on chest X-ray (Figure 1), which supported the diagnosis of coronavirus disease 2019 commonly referred to as COVID-19. Polymerase chain reaction (PCR) confirmed severe acute respiratory syndrome coronavirus 2 (SARS-CoV-2) infection in the patient. 

**Table 1:** Laboratory Results on Admission WBC- White Blood Cells; AST- Aspartate Aminotransferase; ALT- Alanine aminotransferase; LDH- Lactate Dehydrogenase

Laboratory Results on admission			
WBC Count	7.4 (4.8-10.8 k/ul)	Albumin	3.4 (3.4-4.8 g/dl)
Hemoglobin	17.9 (12.0-16.0 g/dl)	Bilirubin, Total	0.3 (0.2-1.2 mg/dL)
Hematocrit	52.9 (42.0-51.0 %)	Bilirubin, Direct – Conjated	0.2 (0.0-0.3 mg/dL)
Platelet	221 (150-400 k/ul)	Alkaline Phosphatase	93 (56-119 unit/L)
Neutrophil %	76.7 (40.0-70.0 %)	AST	93 (9-48 unit/L)
Lymphocyte %	16.8 (20.0-50.0 %)	ALT	47 (5-40 unit/L)
		Total Protein	7.0 (6.0-8.5 g/dl)
Sodium	134 (135-145 mEq/L)		
Potassium	5.8 (3.5-5.0 mEq/L)	LDH	642 (100-190 unit/L)
Bicarbonate	18 (24-30 mEq/L)		
Chloride	96 (98-108 mEq/L)	C Reactive Protein	139.80 (<=5.00- mg/L)
Glucose	116 (70-120 mg/dL)		
Blood Urea Nitrogen	27 (8-26 mg/dL)	D-Dimer Assay	658 (0-230 ng/mL)
Creatinine	2.0 (0.5-1.5 mg/dL)		
Calcium	8.0 (8.5-10.5 mg/dL)	Ferritin	1910 (13-150 ng/mL)

**Figure 2:**
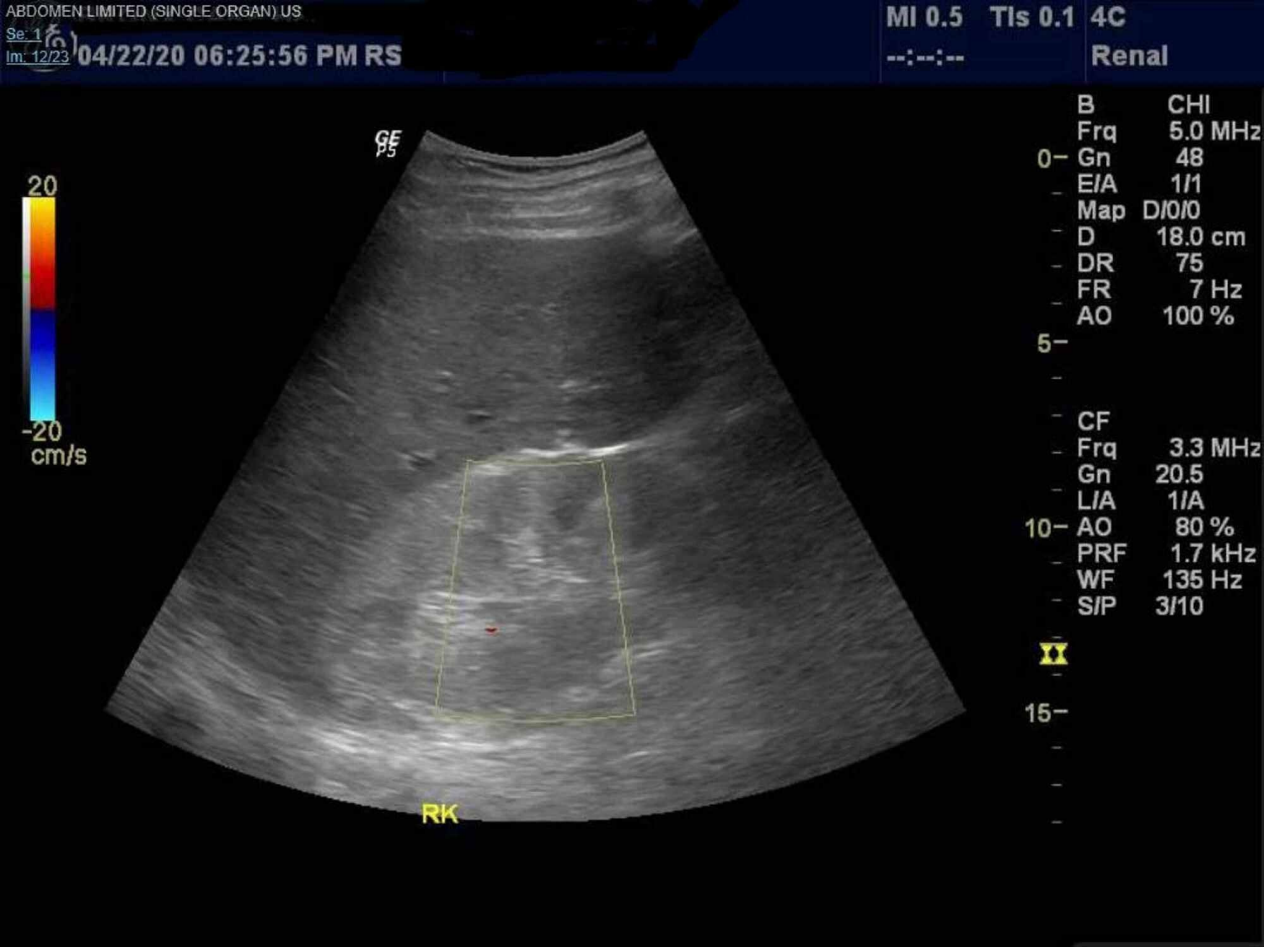
Renal Ultrasound

**Figure 3:**
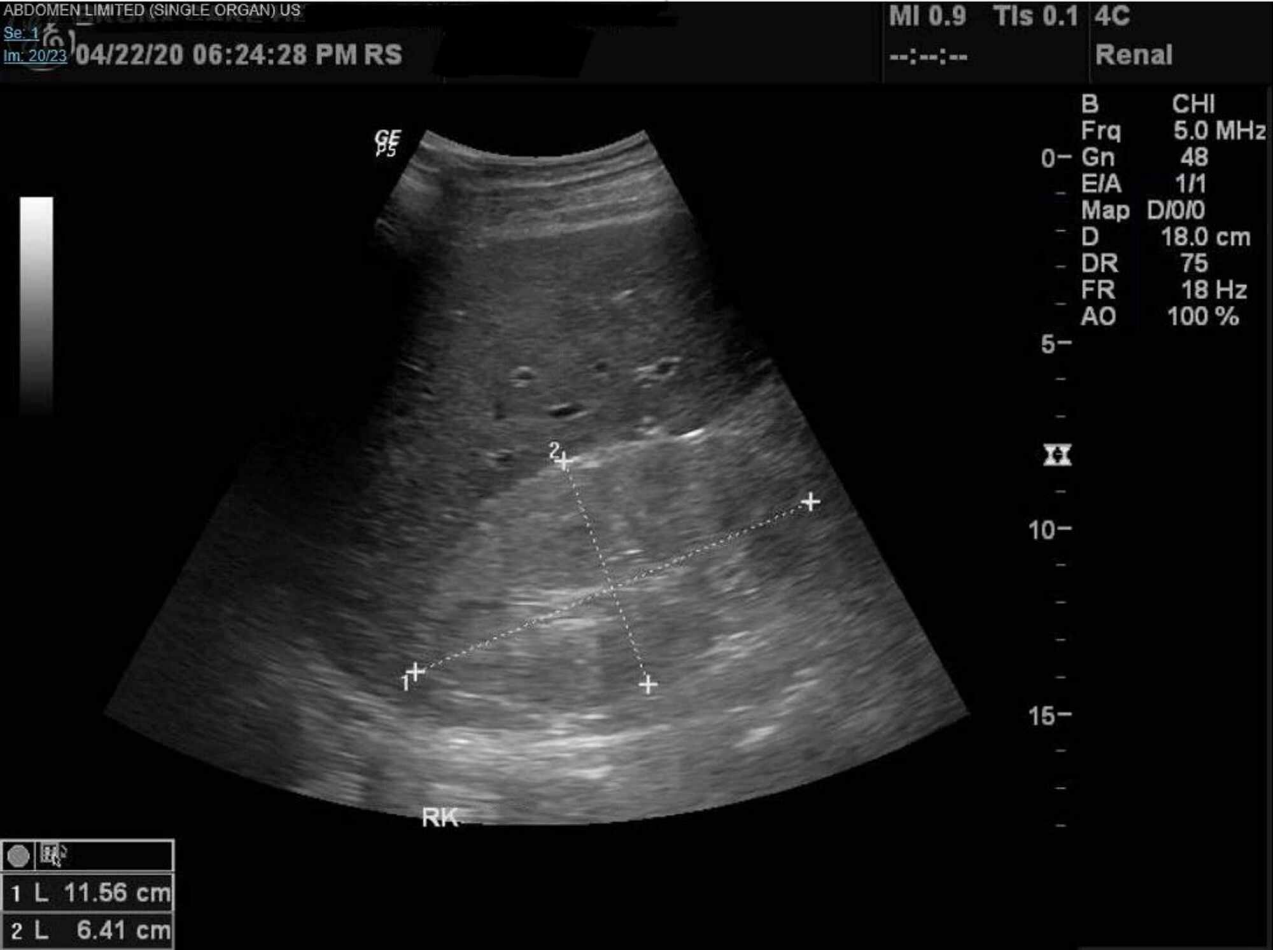
Renal Ultrasound - Right kidney

**Figure 4:**
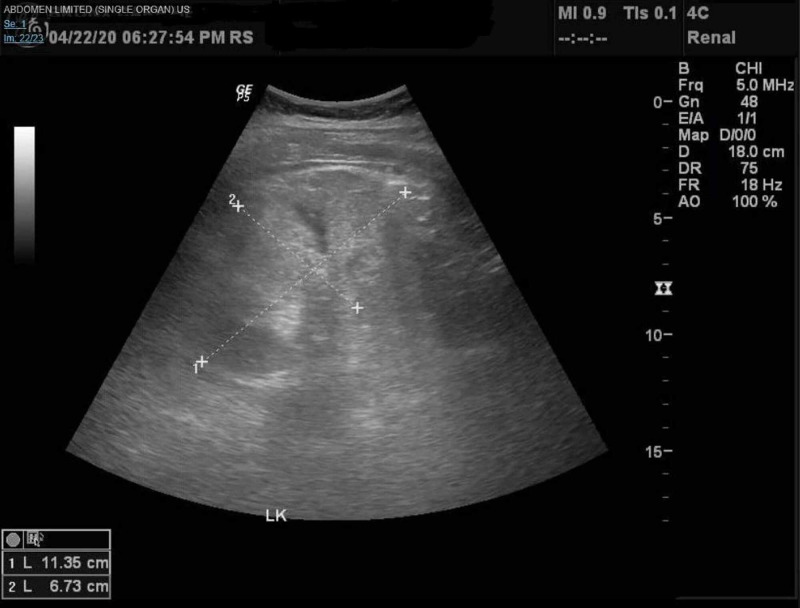
Renal Ultrasound - Left kidney

The patient was placed on antibiotics (Amoxicillin-Clavulanate, Azithromycin, Hydroxychloroquine and Oseltamivir) as well as supplemental oxygen as needed as per hospital COVID-19 protocol, after which he did have significant improvement in his respiratory status. During the admission, a decline in renal function was noted with rising Blood Urea Nitrogen/ Creatinine (BUN/Cr) levels peaking at 100mg/dl and 10.2mg/dl respectively, anion gap metabolic acidosis, and hyperkalemia of 6.1. Renal ultrasound at the time, showed a right and left kidney of 11.6cm and 11.4cm in length respectively, with no hydronephrosis and increased renal cortical echogenicity (Figure 2-4).

In light of patient’s oliguric renal failure and worsening metabolic derangements, the decision was made to start the patient on hemodialysis. Patient’s urine output subsequently increased, electrolytes were stable, and serum creatinine was improving off dialysis. Patient was then discharged to complete a 14-day quarantine at home.

About two months later, the patient was readmitted due to a worsening productive cough and associated shortness of breath. Patient complained of worsening dyspnea that was present since the initial diagnosis of COVID 19. Physical examination of cardiac and respiratory systems showed no abnormalities although noted to have + 1 bilateral pedal edema. Laboratory values on readmission are presented in Table 2. Transthoracic echocardiogram showed an ejection fraction within the normal range (56%) ruling out possible heart failure. Initial lab tests performed also revealed hypoproteinemia, hypoalbuminemia and proteinuria. The urine protein and creatinine ratio confirmed nephrotic range proteinuria of 14865 mg/g. human immunodeficiency virus (HIV) titers, Hepatitis B and C titers, antineutrophil cytoplasmic antibodies (ANCA) vasculitis markers, antinuclear antibody (ANA) markers, and anti-glomerular basement membrane (anti-GBM) antibodies were all negative. C3 and C4 level were normal.

**Table 2:** Laboratory Results on Readmission WBC- White Blood Cells; AST- Aspartate Aminotransferase; ALT- Alanine aminotransferase; LDH- Lactate Dehydrogenase; proBNP- pro B-type natriuretic peptide

Laboratory Results on readmission			
WBC Count	7.4 (4.8-10.8 k/ul)	Albumin	2.4 (3.4-4.8 g/dl)
Hemoglobin	17.9 (12.0-16.0 g/dl)	Bilirubin, Total	0.0 (0.2-1.2 mg/dL)
Hematocrit	52.9 (42.0-51.0 %)	Bilirubin, Direct – Conjugated	0.0 (0.0-0.3 mg/dL)
Platelet	221 (150-400 k/ul)	Alkaline Phosphatase	115(56-119 unit/L)
Neutro %	76.7 (40.0-70.0 %)	AST	19 (9-48 unit/L)
Lymphocyte %	16.8 (20.0-50.0 %)	ALT	20 (5-40 unit/L)
		Total Protein	4.8 (6.0-8.5 g/dl)
Sodium	144 (135-145 mEq/L)		
Potassium	3.2 (3.5-5.0 mEq/L)	ProBNP	1330
Bicarbonate	21 (24-30 mEq/L)		
Chloride	113 (98-108 mEq/L)	C Reactive Protein	15.34 (<=5.00- mg/L)
Glucose	86 (70-120 mg/dL)		
Blood Urea Nitrogen	33 (8-26 mg/dL)	D-Dimer Assay	587 (0-230 ng/mL)
Creatinine	3.4 (0.5-1.5 mg/dL)		
Calcium	6.9 (8.5-10.5 mg/dL)	Ferritin	660 (13-150 ng/mL)

A serum protein electrophoresis (SPEP) was performed and demonstrated monoclonal band proteins confirmed to be immunoglobulin G (IgG) kappa chains on immunofixation and an elevated kappa/lambda ratio of 1.95. A repeat SPEP showed similar results with a worsening kappa/lambda ratio.

A renal biopsy was done to determine the etiology of the nephrotic syndrome with samples outsourced for testing. The patient was managed with calcium channel blockers for blood pressure control and statins. He remained non-oliguric but with slowly worsening serum creatinine levels. Renin-angiotensin-aldosterone system (RAAS) modulation with Angiotensin-converting enzyme (ACE) inhibitors was deferred due to risk of worsening renal function and hyperkalemia and he was discharged to the Nephrology clinic for follow up of biopsy results. Results of the renal biopsy performed ultimately revealed a pattern of collapsing focal segmental glomerulosclerosis (FSGS), in the setting of COVID-19 infection.

**Figure 5:**
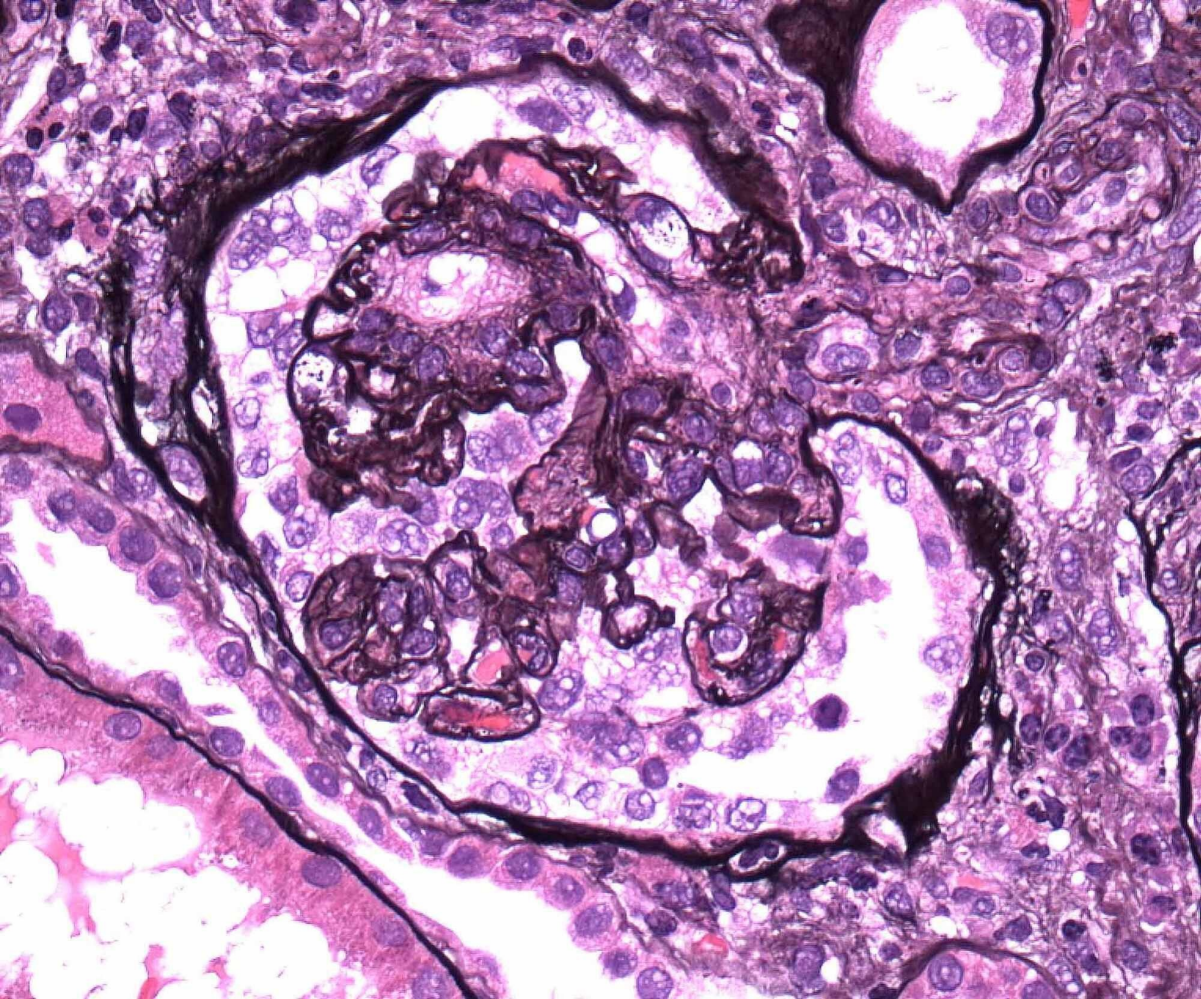
Collapsing Focal segmental glomerulosclerosis

**Figure 6:**
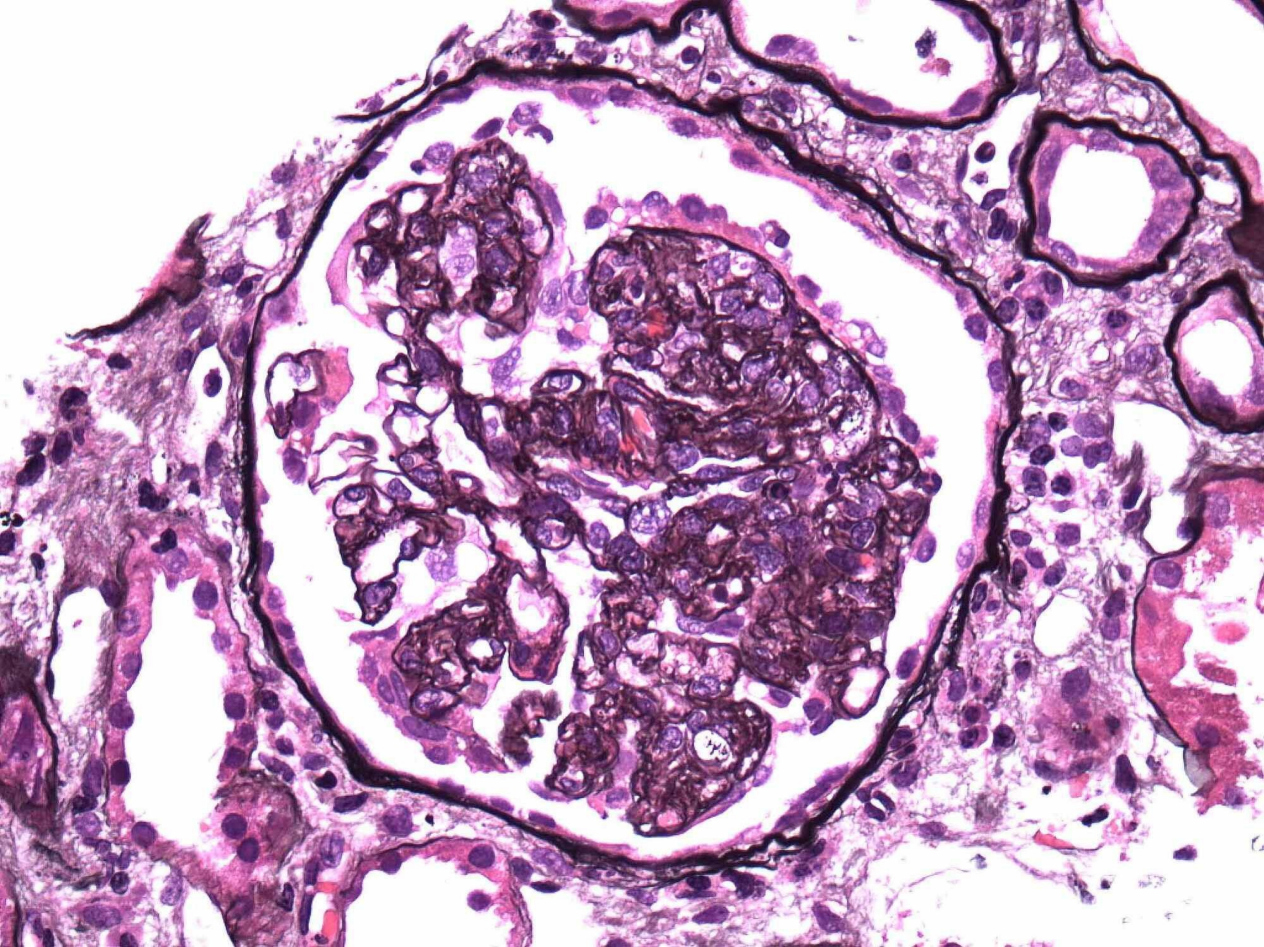
Focal segmental glomerulosclerosis

**Figure 7:**
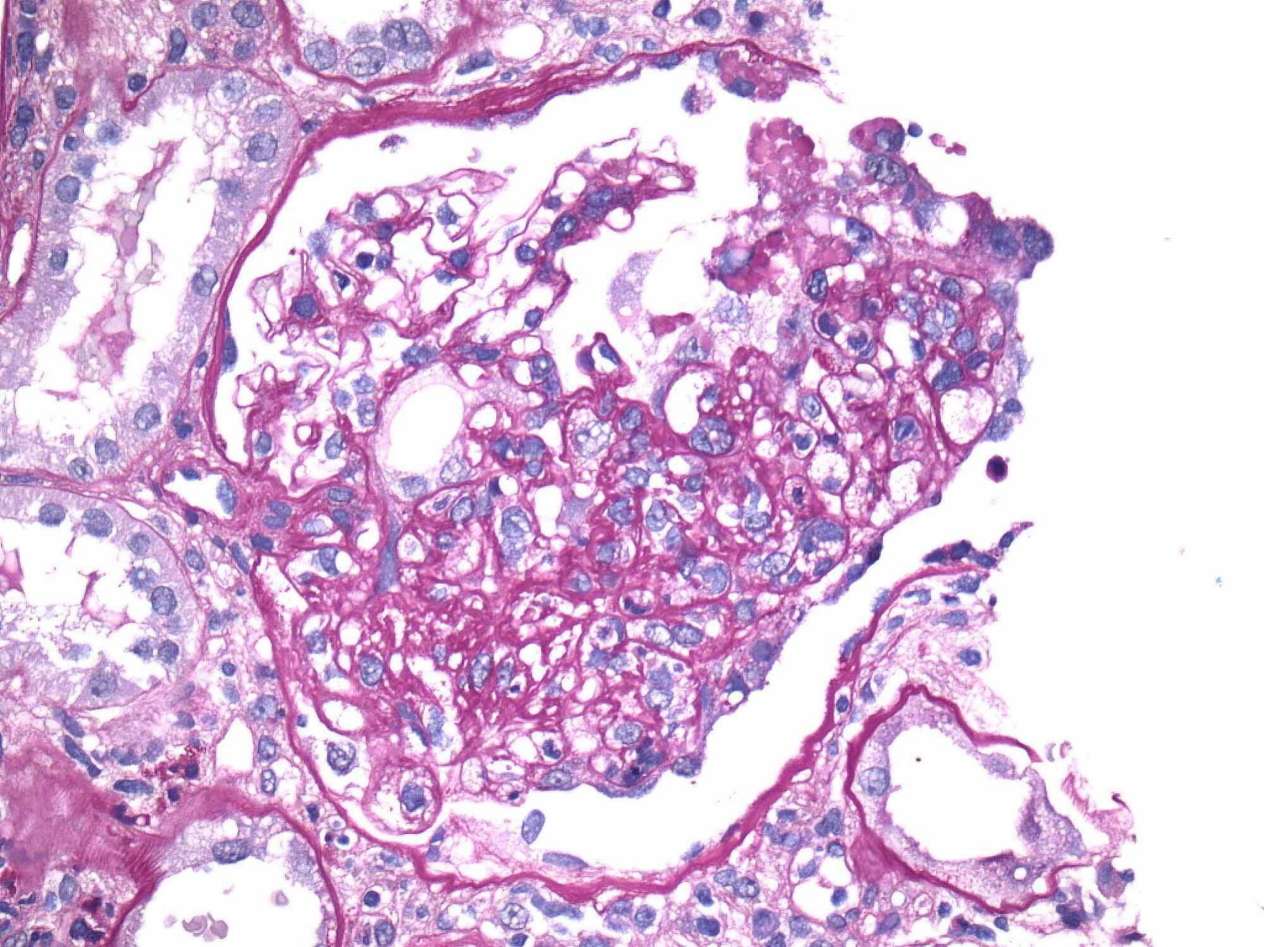
Focal segmental glomerulosclerosis

**Figure 8:**
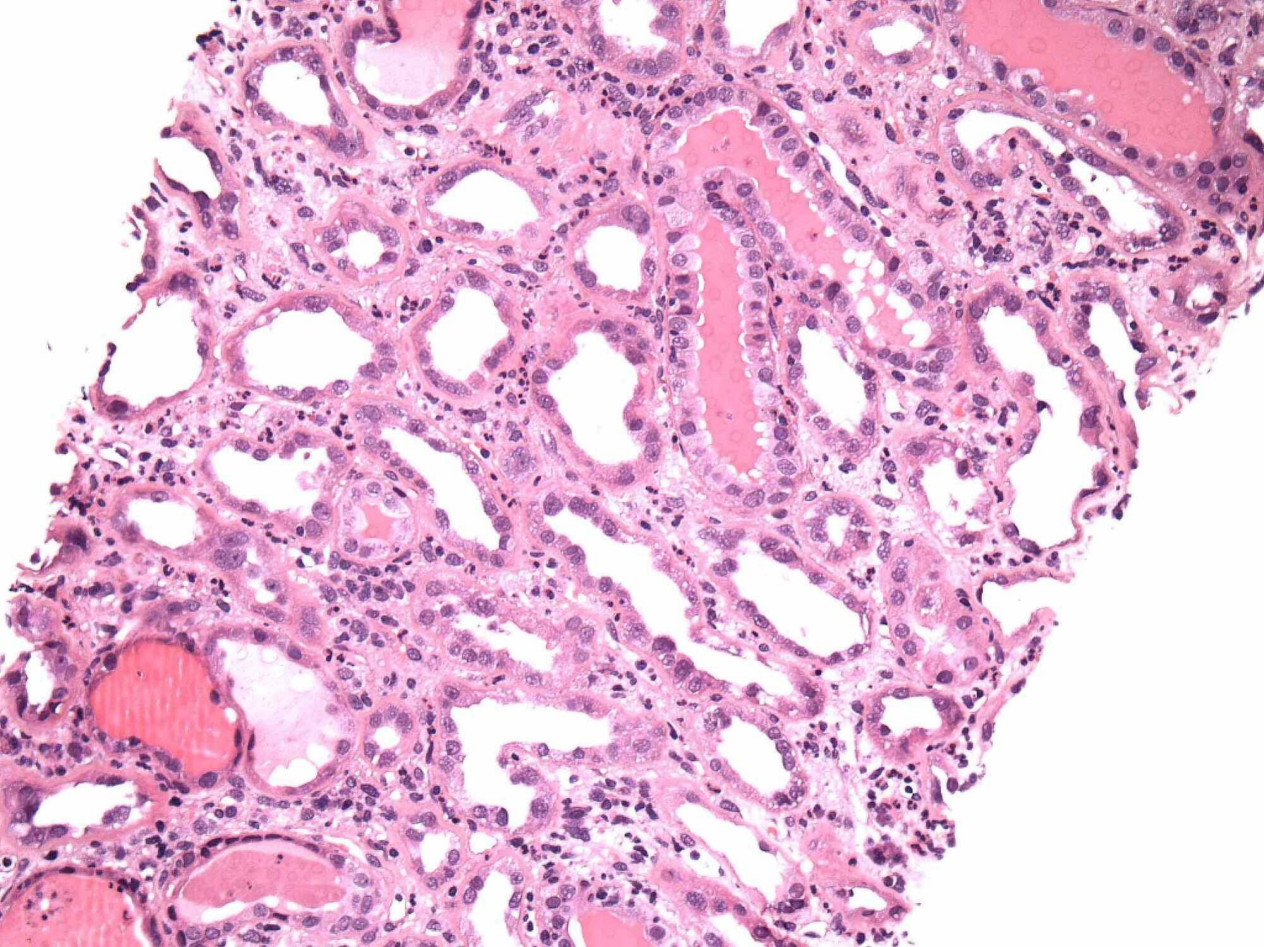
Acute Tubular Injury

**Figure 9:**
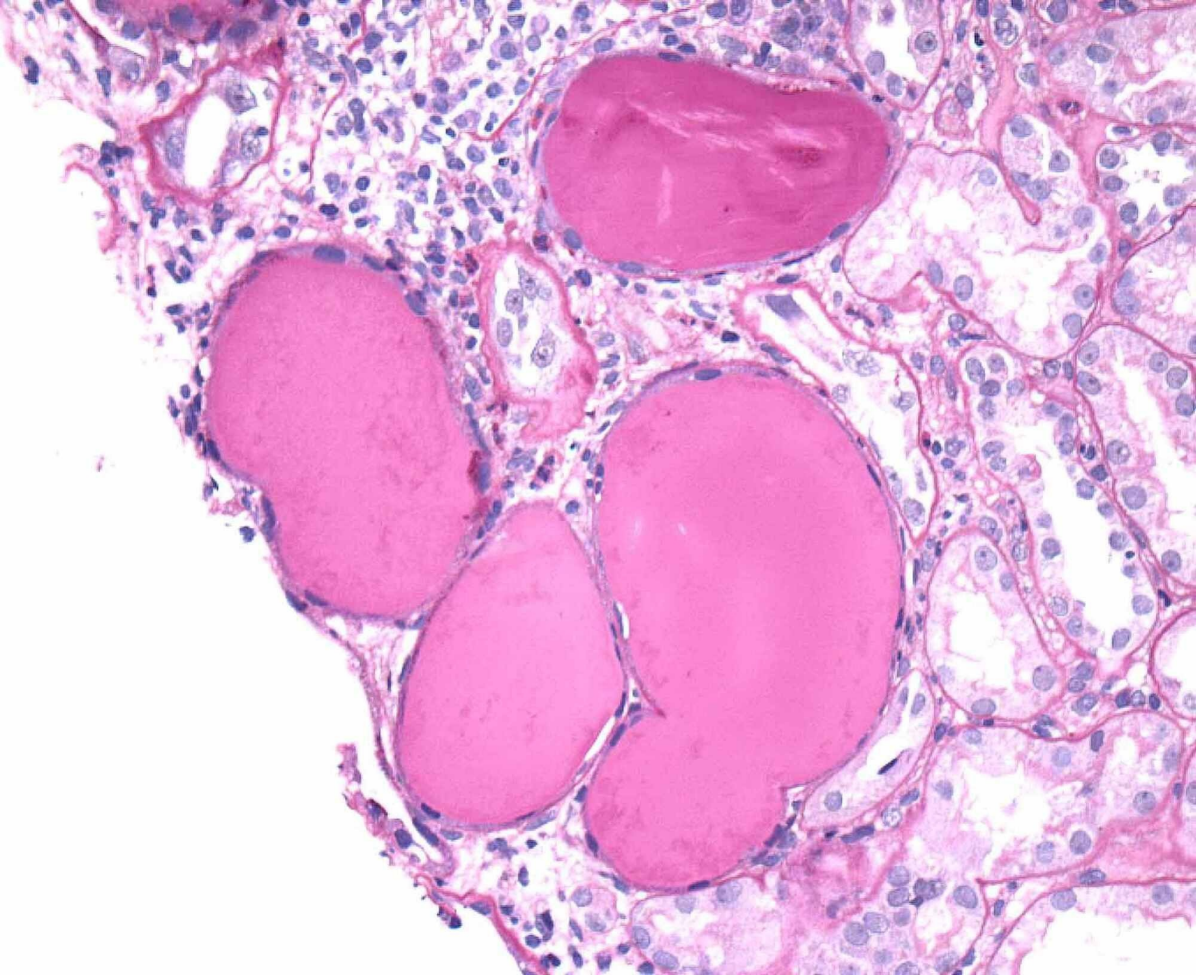
Tubular Microcyst

Renal biopsy showed collapsing focal segmental glomerulosclerosis, diffuse tubular degenerative changes, tubular atrophy, interstitial fibrosis and moderate interstitial inflammation and moderate to severe arteriosclerosis and arteriolosclerosis. Images from electron microscopy are shown below (Figure 5-9). Subsequent immunofluorescence report can be seen in Table 3.

**Table 3:** Immunofluorescence report IgG- immunoglobulin G; IgM- immunoglobulin M; IgA- immunoglobulin A; C3- complement component 3; C1- complement component 1; FBGN- fibrinogen; ALB- albumin

IMMUNOFLUORESCENCE REPORT				
	GLOMERULI	TUBULES	INTERSTITIUM	VESSELS
IgG	8 glomeruli neg (+8 sclerosed)	Negative	Negative	Negative
IgM	3 of 8 glomeruli +/- Segmental tuft	Negative	Negative	Negative
IgA	8 glomeruli negative	Casts 1-2+	Negative	Negative
C3	5 of 8 glomeruli 1+ Segmental to global tuft	Droplets 1+	Negative	Vessel wall 1+
C1	5 of 8 glomeruli 1+ Segmental to global tuft	Negative	Negative	Negative
FBGN	8 glomeruli negative	Negative	Negative	Negative
ALB	8 glomeruli negative	Droplets 1+	Negative	Negative
KAPPA	8 glomeruli negative	Droplets 1+ Casts 1-2+	Negative	Negative
LAMBDA	8 glomeruli negative	Droplets 1+ Casts 1-2+	Negative	Negative

The patient followed up in the nephrology clinic one month later with elevated blood pressure, however his creatinine and estimated glomerular filtration rate (e-GFR) levels were stable. His blood pressure medications were optimized, but his poor prognosis, likely progression to End-Stage Renal Disease (ESRD) and eventual need for renal replacement therapy was discussed with the patient.

## Discussion

Acute renal failure in coronavirus disease 2019 (COVID-19) infection is associated with higher morbidity, mortality, need for renal replacement therapy and worse overall outcomes. According to one New York Hospital, the incidence of acute kidney injury in hospitalized COVID-19 patients was 56.9%. It was also noted that the incidence of acute kidney injury in black patients was 26% greater than Caucasians [5].

Focal segmental glomerulosclerosis (FSGS) is one of the leading causes of high-grade proteinuria with end-stage renal disease (ESRD). There are different histologic variations of FSGS, including classic, collapsing, tip, perihilar, and cellular types. Of all the variants, collapsing FSGS carries the worst prognosis [6]. Collapsing FSGS is associated with a more severe nephrotic syndrome and worse renal outcome than other variants of FSGS. The incidence of ESRD with collapsing FSGS is approximately 73.7% compared to 28.2% in non-otherwise specified FSGS. Risk factors with collapsing FSGS, which determine a worse overall outcome include African ancestry, severe proteinuria, lower estimated glomerular filtration rate (eGFR) at baseline [7].

Many viral infections can cause FSGS including human immunodeficiency virus 1 (HIV-1), cytomegalovirus, parvovirus B19 and Epstein-Barr virus [8,9]. Histologically, the collapsing variant shows segmental or global mesangial consolidation and loss of endocapillary patency in association with extra-capillary epithelial hypertrophy and/or proliferation [8]. The postulated mechanism of podocyte injury due to viral infections is thought to be via direct podocyte toxicity resulting in dysregulation of the cytoskeleton. In HIV-associated FSGS, there is direct infection of the podocyte that results in this dysregulation and cellular phenotype leading to apoptosis [10]. Additionally, in parvovirus infection with collapsing FSGS, parvovirus has been identified within the podocyte, but it is unclear how parvovirus causes podocyte dysfunction. Acute cytomegalovirus infection has also been associated with collapsing FSGS. It is suggested that acute local renal interferon response is responsible for FSGS lesion rather than the direct effect of the virus on the podocyte [11]. Though the exact mechanism of podocyte injury for COVID-19 infection has not been determined, given the inflammatory milieu involved, it can be postulated that podocyte dysregulation likely occurs due to a cytokine storm. This, in turn, causes a massive release of interferons, granulocyte colony-stimulating factor, and interleukins which can cause a direct kidney injury [12]. Though coronavirus was not identified in the podocyte, given the mechanism behind other viral infections, there may be a direct infection of the podocyte causing apoptosis and dysregulation of the cytoskeleton.

According to recent data, the incidence of podocytopathy and proteinuria is higher in patients of African descent [5,12]. Studies have shown that the Apolipoprotein L1 (APOL1) gene is expressed exclusively in individuals of African descent. Patients with APOL1 gene have higher baseline proteinuria and kidney disease progression [6]. APOL1 is an under-recognized cause of FSGS leading to ESRD. Approximately 40% of ESRD is attributed to FSGS in black, of which 72% is associated with APOL1 genetic variants. Also, the effect of HIV on podocytes is strongest in individuals with two APOL1 risk alleles. The odds of developing HIV associated nephropathy is 29-fold higher in patients with high-risk alleles [6,8].

Recent studies show that the mechanism of acute kidney injury with SARS-CoV-2 infection is variable and not well understood. Santoriello et al. [13] performed postmortem kidney biopsies on 42 patients and found that acute tubular injury was the main pathologic finding correlating with a history of acute kidney injury. Kudose et al. [12] performed kidney biopsies on 17 patients with COVID 19 infection, of which 29% had collapsing FSGS as the primary diagnosis for acute kidney injury. Of these 17 patients, 70% had acute tubular injury seen on pathology.

## Conclusions

In conclusion, collapsing FSGS is an under-recognized cause for renal failure associated with SARS-CoV-2 infection. Acute renal failure with SARS-CoV-2 infection leads to higher mortality and worse prognosis. There should be a low threshold to suspect collapsing FSGS in patients with high-grade proteinuria, acute kidney injury, African descent and nephrotic syndrome in patients with SARS-CoV-2 infection. Additionally, APOL1 genetic testing should be done in patients of African descent with high-grade proteinuria as the progression of renal disease worse in patients with high-risk allele. Further research needs to be done on the mechanism of injury to the podocyte and potential therapies for early intervention.
